# Influence of Agro-Industrial By-Products Inclusion on Growth Parameters and Carcass Quality in *Ovella Galega* Lambs

**DOI:** 10.3390/ani16060921

**Published:** 2026-03-15

**Authors:** Aurora Cittadini, Roberto Bermúdez, Vasco Cadavez, Adriana González-Peaguda, Raúl Bodas, José Manuel Lorenzo

**Affiliations:** 1 Centro Tecnológico de la Carne de Galicia, Avd. Galicia No. 4, Parque Tecnológico de Galicia, San Cibrao das Viñas, 32900 Ourense, Spain; auroracittadini@ceteca.net (A.C.); robertobermudez@ceteca.net (R.B.); adrianagonzalez@ceteca.net (A.G.-P.); 2 CIMO, LA SusTEC, Instituto Politécnico de Bragança, Campus de Santa Apolónia, 5300-253 Bragança, Portugal; vcadavez@ipb.pt; 3 Agrarian Technological Institute of Castille and Leon (ITACyL), Ctra. Burgos km 119, 47071 Valladolid, Spain; bodrodra@itacyl.es; 4 Área de Tecnología de los Alimentos, Facultad de Ciencias de Ourense, Universidade de Vigo, 32004 Ourense, Spain

**Keywords:** brewers’ grain, grape pomace, olive-cake, Galician lamb, productive parameters, carcass attributes, carcass morphology, commercial cutting

## Abstract

This study investigated the impact of including 10% of dried brewers’ grain (BG), grape pomace (GP), or olive cake (OC) in the finishing diets of thirty-two *Ovella Galega* lambs on their growth parameters and carcass quality. Productive parameters such as live weight, average daily gain (ADG), and subcutaneous fat depots were monitored. The following carcass traits were also evaluated: carcass weight, conformation, fatness degree, morphology, and pH. Moreover, the left-half carcasses were sectioned into the main commercial cuts. Results showed that the type of diet did not significantly affect (*p* > 0.05) the growth performance and carcass characteristic of the animals. All groups reported similar (*p* > 0.05) live weights, ADG, and pre-slaughter fat thickness. In the same manner, the lack of significant variations observed in the productive parameters was also reflected in the carcass traits, showing comparable (*p* > 0.05) weights, dressing percentages, conformation, fatness levels, morphometric measures, and pH among treatments. Furthermore, the commercial value of the animals was not compromised (*p* > 0.05) by the experimental treatments. Thus, these outcomes suggest that the employment of these agri-food by-products, at moderate levels, could represent a viable and sustainable feeding approach for these autochthonous lambs.

## Simple Summary

Today, the growing food demand has intensified the generation of agri-food by-products, which are frequently inefficiently managed, posing environmental and economic issues. Brewers’ grain, grape pomace, and olive cake represent some of the most abundant residues generated by the Spanish agro-industrial sector and have revealed a high potential as alternative feed ingredients. However, there is scarce information on their use in lamb nutrition, especially in autochthonous breeds, such as the *Ovella Galega*. In this context, our study evaluates how replacing a 10% of the finishing concentrate with these three by-products impacts the productivity and carcass quality of these local lambs. Furthermore, considering the limited number of investigations on Galician sheep as a meat producer, this work represents a starting point to enhance its handling. Data showed that the incorporation of these brewery, winery, and olive oil by-products in the finishing diets did not negatively affect the growth parameters and carcass traits of these animals. In this sense, this alternative dietary strategy could be considered a viable and sustainable approach for the production system of these local lambs, along with a promising solution to reduce food waste and boost the circular economy, aligned with the current sustainability policies.

## 1. Introduction

Nowadays, sustainability is driving a multidimensional transformative process reshaping governance frameworks, consumption habits, and production systems, including the agri-food sector, particularly in response to the necessity of feeding a growing population, while mitigating climate change and preserving natural resources [1,2,3]. According to the United Nations’ report [4], the global population is in fact projected to reach nearly 10.3 billion people by the mid-2080s, thereby intensifying the demand for food and agricultural resources. Nevertheless, approximately one third of the food produced worldwide (more than 1.1 billion tons per year), is lost or wasted [5,6]. At the same time, the agro-industrial sector generates annually immense amounts of by-products that frequently remain underutilized despite their potential economical and nutritional value [5]. In this context, the valorization of agri-food by-products has emerged as crucial strategy to enhance resource efficiency, and to promote circular economy principles through their reutilization within the food chain [7,8].

In Spain, the brewing, wine, and olive oil industries are among the main sources of agro-industrial residues, with brewers’ grain (BG), grape pomace (GP), and olive cake (OC) being some of the most plentiful by-products generated [3,9]. Spain is in fact the third greatest European beer producer, with annually over 4.1 million tons, which results in the generation of approximately 826,000 tons of brewers’ grain [3,9,10]. In addition, at global level, this country occupies the third position among the most important wine-producing nations, processing around 4.5 million tons of grapes, of which 20–25% is wasted as grape pomace [9,10,11]. Moreover, the Spanish olive oil industry represents the world’s leading producer, utilizing around 4.6 million tons of olives annually and generating a large amount of olive cake (around 3.7 million tons), which can represent up to 80% of the processed olives’ weight [9,10,12]. Furthermore, these three by-products are rich in fiber, protein, lipids, and bioactive compounds such as polyphenols and unsaturated fatty acids (especially oleic acid), which have been associated to potential benefits on animal health and productive performance, and as a consequence, on the derived products quality [12,13,14].

On the other hand, in the livestock sector, the rise of feed costs, the increasing competence between animal and human nutrition, as well as demand for more sustainable and resilient food systems, highlight the need of identifying alternative ingredients able to improve the farm sustainability and profitability without compromising the productivity and the quality of the final product [7,15]. In this context, in the Mediterranean regions, where raising small ruminants (such as lambs) as livestock is widely practiced, the use of agro-industrial by-products in livestock feeding has gained increasing attention in the context of sustainable production systems [8]. However, there is still scarce information about their influence on ovine productive traits and carcass characteristics, especially for local lamb breeds. *Ovella Galega* is a threatened or endangered autochthonous lamb breed from Galicia (northwestern Spain), counting a total of 5157 registered animals [16], characterized by a small size and generally raised in extensive or semi-extensive production systems. Nonetheless, to date, there is a dearth of studies about its productive and carcass quality, as well as lack of scientific investigations evaluating the impact of agro-industrial by-products as alternative dietary ingredients on these animals [17,18,19,20].

Therefore, the objective of this study was to evaluate the effects of incorporating brewers’ grain, grape pomace, and olive cake as partial (10%) feed replacers in Galician lambs, focusing on their growth parameters and carcass quality traits. An inclusion level of 10% was defined based on the previous literature findings and considering the animals’ nutritional requirements [21], ensuring a moderate substitution and a balanced comparison among treatments, while minimizing potential negative impacts from excessive fiber, lipid content, or secondary compounds like tannins. Furthermore, to our knowledge, this is the first investigation assessing the use of these agro-industrial by-products in this autochthonous breed. Accordingly, we hypothesized that a 10% dietary supplementation with these three by-products would not impair/compromise the animals’ growth performance or carcass characteristics compared to a conventional diet. Hence, this work provides innovative scientific evidence to support the valorization of local agri-food by-products as well as contributes to the development of more sustainable feeding strategies for ovine production systems, with a particular emphasis on endangered lamb breeds.

## 2. Materials and Methods

This study was carried out in the facilities of the experimental farm of the Ourense Institute of Economic Development (INORDE), in Armariz, Nogueira de Ramuín, Ourense (northwestern Spain), located at 42°22′17.43″ N latitude, 7°43′12.73″ W longitude, and at an altitude of approximately 510 m above sea level. The experimental procedures employed in this study were approved by the Institutional Animal Care and Use Committee of the Agrarian Technological Institute of Castilla and León (Spain) under the protocol number 2025/09/CEEA.

### 2.1. By-Products and Feeds Formulations

Brewers’ grain, red grape pomace, and olive cake were kindly provided by three Galician agro-industrial facilities: González Carballal SL—Cerveza Esmorga (Untes, Ourense, Spain), Bodegas Gómez Sanmartín S.L.—BODEGOSA (Castrelo de Miño, Ourense, Spain), and Ouro de Quiroga S.L. (Quiroga, Lugo, Spain), respectively. Each by-product was carefully dried (<10% moisture) and successively was delivered to a registered commercial feed manufacturer, Cecoagro Central de Compras, S.L. (Begonte, Lugo, Spain), where they were ground and chemically characterized (Table 1).

Four different feeds were elaborated: a control (CON) consisting of a commercial concentrate, and three by-product-based feeds—brewers’ grain (BG10), grape pomace (GP10), and olive cake (OC10) feeds—where 10% of the traditional concentrate was replaced with the corresponding dried agro-industrial by-product. The concentrates were ground to prevent selective feeding of ingredients by lambs and to facilitate their distribution. The feed formulations and composition are presented in Table 2, with ingredients expressed in g/kg of feed.

Samples of the by-products and formulated concentrates were analyzed for dry matter, ether extract, crude protein, ash, and crude fiber according to the Association of Official Analytical Chemists procedures [22]. Neutral detergent fiber (NDF) and acid detergent fiber (ADF) were determined following the method of Van Soest et al. [23].

### 2.2. Experimental Design and Animal Management

Thirty-two weaned *Ovella Galega* lambs, with approximately 57 days of age and initial average live weight of 7.09 ± 1.12 kg, were divided into four homogeneous groups (n = 8 per group) and submitted to the four different dietary treatments: one group was fed a control concentrate (CON), and the other three groups of animals were finished with the experimental concentrates containing 10% of brewers’ grain (BG10), grape pomace (GP10), and olive cake (OC10), respectively. All animals were dewormed, individually ear tagged, and housed in four separate pens. Considering that it is a pilot study and it is the first essay evaluating the inclusion of these agro-industrial by-products in the finishing diets for *Ovella Galega* lambs, feeding management was established in accordance with the commonly used practices at the experimental farm where the trial was conducted, as well as in order to avoid imposing abrupt or unfamiliar management conditions on this local breed. The ground concentrates were offered once daily (at 9:00 h) at a restricted level (32 g concentrate/kg body weight [24]). In addition to the experimental feeds, lambs had *ad libitum* access to clean water, lick mineral blocks, and hay (separated feeding troughs) over the whole trial. Hay (DM: 89.56%, EE: 1.39% DM, CP: 5.18% DM, Ash: 5.81% DM, CF: 31.72% DM, ADF: 46.42% DM, NDF: 66.78% DM, Ca: 284.71 mg/100 g, P: 220.88 mg/100 g) originated from mown permanent pastures, typically composed of a natural mixture of grasses and legumes. Before starting the trial, each lot was adapted to the experimental dietary treatments for 7 days. After this, all animals were fattened to achieve the slaughter weight of 12 ± 1.00 kg, which is the value accepted by the local market for *Ovella Galega* lambs. Thus, the growth essay for all animals lasted around 64 days.

As regards animal productivity, live body weight of lambs was registered at the beginning of the study (IBW) and at slaughtering (BWS), monitored every 15 days during the whole experimental period. The weighing was carried out at the same time of the day (after overnight fasting of feed and water) with the objective of minimizing the effect of normal daily variability on measurements. Moreover, growing rates were assessed. The total average daily gain (ADG) was calculated as the regression coefficient (slope) of body weight against time.

In parallel, the ultrasonography assessment of subcutaneous fat thickness (SFT) was carried out to evaluate the amount and distribution of fat in vivo as well as to assess the ability of this method to predict potential phenotypic traits that could later be found in the animal carcass [25,26]. In particular, the subcutaneous fat depots were evaluated at the beginning and the end of the trial (the day before slaughtering) following the indications reported in previous studies [27,28]. SFT measures were realized via B-mode with a portable ultrasonographic imaging system Mindray Z60 equipped with a L14-6P linear transducer set at 10 MHz (30 × 8 mm) (Shenzhen Mindray Bio-Medical Electronics Co., Ltd., Shenzhen, China). All assessments were carried out by the same technician and images were taken on the left side, at the level of the 3rd–4th lumbar vertebrae of the lambs. Ultrasound gel was used as a coupling medium in the zone of interest, and the probe was placed perpendicular to the backbone for the evaluation. The transducer was applied with minimal pressure to avoid compression of fat and muscle tissues [25]. When a satisfactory image was obtained, it was recorded for later analysis. All ultrasound measurements were carried out employing ImageJ 1.54g software (National Institute of Health, Bethesda, MD, USA; http://imagej.org accessed on 2 October 2025) by the same trained researcher to prevent measurement technique variability. The fat depth was measured including the skin [27].

### 2.3. Slaughtering and Carcass Measurements

After around 64 days (reaching the required body weight), animals were transported to a commercial abattoir, Magefrigor S.L. (Ourense, Spain), located 18.8 km away from the experimental farm, the day before the slaughter. Lambs had a 16 h solid fasting period, after which all lambs were weighed to assess the final live body weight (BWS). Successively, all animals were slaughtered, at a mean age of 122 days and with an average live weight of 12.82 ± 1.03 kg, following the standard commercial practices involving head electrical stunning and severing the carotid arteries and jugular veins, according to EU legislation [29]. Following slaughter, the carcasses were weighed to record the hot carcass weight (HCW). Then, carcasses were chilled in a cold chamber (4 °C) for 24 h and successively weighed again to register the cold carcass weight (CCW). These data were used to calculate the hot carcass yield (HCY), also known as real dressing percentage, and the cold carcass yield (CCY), commercial dressing percentage, employing the following formulas: HCY = (HCW/SBW) × 100 and CCY = (CCW/SBW) × 100, respectively [30].

Furthermore, 24 h post mortem officially accredited slaughterhouse personnel classified carcasses on the base of their conformation and fatness degree following the methodology routinely applied in this local abattoir [31], under standard commercial conditions. This evaluation is considered a potential tool to categorize and predict the animal carcass quality. In particular, each carcass was graded following the SEUROP conformation system using its full (completely) subdivided 18-point scale ranging from 1 (very bad conformation, P-) to 18 (very good conformation, S+) including the following subclasses: P−, P, P+, O−, O, O+, R−, R, R+, U−, U, U+, E−, E, E+, S−, S, S+. In addition, fatness evaluation was performed considering the amount and distribution of fat in the external and internal parts of the carcass, and the complete 15-point scale was employed, from 1 (low fat, 1−) up to 15 (very high fat, 5+), including the corresponding subcategories: 1−, 1, 1+, 2−, 2, 2+, 3−, 3, 3+, 4−, 4, 4+, 5−, 5, 5+.

In addition, for an objective assessment of the carcass conformation, the following carcass morphometric parameters were measured [32]: external carcass length (ECL), leg length (LL), width of the shoulders (WS), width of the buttocks (WB), anterior circumference of the buttocks (ACB), posterior circumference of the buttocks (PCB), and circumference of thorax (CT). Each carcass was also divided sagittally and sectioned into two symmetric halves, and the internal carcass length (ICL) was evaluated on the left half-carcass [33]. Furthermore, subcutaneous fat thickness (FT) was also determined using a caliper on the medial surface of the left half carcass, in the ventral–caudal region, at the junction between the flank and the hind leg, where subcutaneous fat cover was sufficiently developed and detectable. This location was selected due to the low fatness and typical small size of the carcass of Galician lambs. Indeed, in this light lamb breed, dorsal fat depots can be poorly developed; therefore, this ventral site can provide a distinctive and repeatable assessment among all carcasses. Finally, the carcass compactness index (CCI) was determined applying the following formula: CCI = CCW/ICL [33].

Moreover, pH was measured 24 h post-mortem between the 4th–5th ribs in the *longissimus thoracis et lumborum* muscle of the left half-carcass using a portable pH-meter (Hanna Instruments S.L., Eibar, Gipuzkoa, Spain) equipped with a penetrating electrode and an automatic temperature compensator.

Finally, the left half-carcass was dissected using the cutting protocol routinely employed in our research center, and the following commercial cuts were obtained: leg, ribs, neck, loin, breast, tenderloin, and shoulder. Each meat cut was weighed and its individual yield calculated using the following equation: cut (%) = cut weight/left half-carcass weight. This procedure was carried out to assess the carcass composition and yield, as well as to estimate its potential economic value.

### 2.4. Statistical Analysis

SPSS software (SPSS 26.0, Chicago, IL, USA) was employed to carry out all the statistical analysis in this study. SEUROP conformation and fatness subclasses were numerically coded following their ascending order (1–18 and 1–15, respectively) and treated as quantitative variables for statistical purposes. Shapiro–Wilk and Levene tests were utilized to assess the normal distribution and variance homogeneity, respectively. Then, all data were examined using a one-way analysis of variance (ANOVA), where parameters were included as dependent variables, and the dietary treatment (CON, BG10, GP10, and OC10) was introduced as fixed effect. The following statistical model was applied:

Y_ij_ =μ + D_i_ + ε_ij_

where Y_ij_ is the observed dependent variable, μ the overall mean, D_i_ the dietary treatment effect, and ε_ij_ is the residual random error. Each lamb was considered an experimental unit. The pairwise differences between least-square means were verified using Duncan’s *t*-test. The level of statistical significance was set at *p* < 0.05.

## 3. Results and Discussion

### 3.1. Effect of the Finishing Diet on Productive Parameters and Carcass Traits

The growth parameters of lambs are shown in Table 3. As can be seen, body live weights were not influenced (*p* > 0.05) by the type of dietary treatments. The animals’ initial body weights, on average 7.09 ± 1.12 kg, were not significantly (*p* > 0.05) different among groups, indicating that the groups were balanced at the beginning of the trial. Similarly, the final live weight at slaughtering did not differ significantly among experimental groups, with a mean value of 12.82 ± 1.03 kg. This finding could be expected considering that lambs were allocated into weight-balanced lots at the beginning of the study, as well as being of the same breed, with similar age, and sacrificed at the predetermined weight of 12 ± 1.00 kg, as required by local market. In addition, other authors found that the inclusion of a 10% or comparable amounts of brewers’ grain [33,34,35,36], grape pomace [37,38,39], or olive cake [40,41,42,43] in ovine diets had no significant effects on the BWS of lambs. Considering the live weights obtained in this study, the above cited authors [33,34,35,36,37,38,40,41,42,43] found higher values than ours, though that could be expected considering the characteristic small size and minor growing rates of the Galician breed [18,19,44,45]. Furthermore, feeding management could play a key role. In fact, a previous study [18] reported greater live weights for Galician lambs slaughtered at a similar age (120 days, 20.08 ± 1.80 kg) to those in the present work (around 122 days), fed with conventional concentrate and hay *ad libitum*. This outcome could be attributed to the fact that in the present study animals had a restricted access to concentrate, 32 g/kg body weight, to ensure that it was fully consumed. This amount is smaller than the consumption level reported by other authors [46] when lambs have free access to concentrate (38–40 g/kg body weight). However, our results agree with those reported for 75-day-old *Ovella Galega* lambs (12.91 ± 1.39 kg) reared under a semi-extensive system [19].

Regarding the average daily gain, no statistically significant differences (*p* > 0.05) were detected among treatments. Likewise, initial and final live weights and the duration of the finishing period (64 days) were comparable among groups, with no statistically significant variations observed. This result suggests that partial substitution (10%) of concentrate with BG, GP, or OC did not produce statistically significant differences in the growth of the Galician lambs/lambs’ growth under the conditions of this study. Similarly, other researchers [34,35] have published that including wet brewers’ grain, even at relatively higher substitution levels (up to 35%), in lamb diets did not influence the ADG and overall growth performance. The same tendency was found in previous studies using grape pomace [37,47] or different forms of olive cake [40,43] as partial feed replacers. On the other hand, our data are much lower than those observed in the works employing the three agri-food by-products in intensive systems, showing ADG close or higher than 200 g/d [37,40,43,47,48]. This discrepancy could be explained by the lower growth potential of the *Ovella Galega* breed, as well as low initial body weights. As mentioned above, this local lamb breed is characterized by a small size and not elevated growth rates [18,19,44,45]. Indeed, the average daily gains achieved in this study are in agreement with the range of values (70–150 g/d) registered in 120-day old Galician lambs reared under extensive or semi-extensive systems [18]. Thus, although it is the first time that these experimental feeds were used in this autochthonous breed, it is evident that the feeding management applied in this study favored a satisfactory adaptation of the lambs to these alternative ingredients and avoided negative effects on their growing performances.

**Figure 1:**
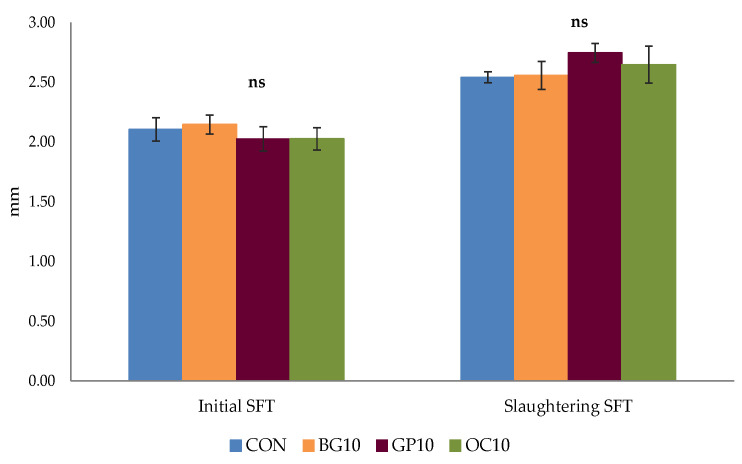
Effect of the dietary treatment on the subcutaneous fat thickness (SFT) from *Ovella Galega* lambs. CON: Control feed group; BG10: Group supplemented with brewers’ grain feed; GP10: Group supplemented with grape pomace feed; OC10: Group supplemented with olive cake feed; ns: Not significant.

As regards the in vivo ultrasonographic measures of the subcutaneous fat depots, as can be seen in Figure 1, no significant differences (*p* > 0.05) were observed in the initial and pre-slaughter values among the four groups. At the beginning of the trial, all animals registered mean values of 2.08 ± 0.05 mm. Before slaughtering, GP10 group showed the highest values (2.75 mm), followed by OC10 (2.65 mm), BG10 (2.56 mm), and CON (2.54 mm), though not significantly (*p* > 0.05). These outcomes suggested that the by-products included in the diets did not affect (*p* > 0.05) the fat deposition pattern of the animals during the finishing period, and all groups showed an increase of approximately 26% compared to the initial value. However, to the best of our knowledge, there are no previous studies on the in vivo ultrasonographic assessment of SFT in Galician lambs, or in other genotypes fed BG, GP, or OC. Actually, earlier investigations on light lambs or sheep finished with these by-products [36,38,42,49,50] assessed this parameter post-mortem, in the carcass or in different anatomical points of the *Longissimus thoracis and lumborum* muscle, as discussed in more detail below. Nevertheless, our SFT values at slaughtering (on average 2.62 ± 0.05 mm) are in accordance with the range of values (0.4–5.1 mm) published on Spanish lambs with slaughtering weight close to ours [51] and measured at the same anatomical level (3^rd^–4^th^ lumbar vertebrae).

The effect of the type of finishing diet on carcass characteristics is shown in Table 3. As can be observed, CCW and HCW values did not differ (*p* > 0.05) among groups, indicating that the use of BG, GP, and OC as alternative dietary ingredients did not produce significant variations on these parameters. This trend was also found in previous investigations including levels of BG even greater than ours (20, 35, 40, 60, and 80%) in the finishing diets of Santa Ines [36] and Polish Merino [34] lambs. Moreover, other authors indicated that carcass weights were not affected by the use of 10% of GP [37,52] or OC [42,43] in the lambs’ nutrition. On the other hand, Chikwanha et al. [53], investigating the effect of feeding graded percentages (0, 5, 10, 15 and 20%) of dried GP on Dohne Merino lambs, found an increase of hot and cold carcass weights, with optimum inclusion levels at 12.2 and 12.1%, respectively. Furthermore, as in the case of growth parameters, the carcass weights registered by the above-mentioned studies [34,37,42,43,52] were greater than ours, though this could be related to various factors such as breed-intrinsic characteristics, age, and dietary management. Actually, our data are in line with those recorded in *Ovella Galega* lambs [18] as well as with the range of values (5.0–8.1 kg) reported for other light Spanish breeds [49].

Statistical analysis indicated that hot and cold carcass yields were not significantly affected (*p* > 0.05) by the type of diet applied, showing mean values of 49.41% and 47.18%, respectively. Other authors [34,36] also did not find significant variations in dressing percentages obtained including BG in comparison with the control group. Similarly, it was observed that replacing the 10% or higher levels (12.5, 20, 25, 30 and 40%) of concentrate with OC did not affect the carcass yields of lambs [40,42,43,54]. The same trend was also found in a recent investigation [37], where Merinolandschaf lambs were fed diets containing 10 or 20% of GP. Meanwhile, a previous study on Dohne Merino lambs revealed that the inclusion of 10, 15, and 20% GP linearly increased their dressing ratios, obtaining better values in the group with greatest level of substitution [53]. Nevertheless, in the present study, the lack of dietary influence on carcass yields suggests that the inclusion of these residues at 10% did not alter the relationship between the live weight, gastrointestinal content, and tissue development in our lambs. This is particularly important for rustic breeds, where their efficiency and ability for meat production could be influenced by the feeding system [18,19,55]. As regards the values recorded in this work, our data were numerically close or higher than those reported in previous studies using the studied agri-food by-products in lamb nutrition [34,36,40,43,56]. In addition, our outcomes are in accordance with data reported by Adan et al. [18] in *Ovella Galega* lambs with similar ages and finished with concentrate and hay indoors, suggesting that our animals had a satisfactory adaptation to the alternative ingredients.

With regard to carcass conformation and fatness degree, no significant differences (*p* > 0.05) among batches were detected, indicating that the inclusion of the studied by-products did not influence these parameters. Other authors [38,40,43] found this same trend replacing 10% of forage or concentrate with GP or OC, respectively, as well as using greater substitution percentages (up to 30% for GP and 40% for OC). Examining the scores obtained in the cited investigations, their values cannot be easily compared since we employed broader classification scales. According to our methodology, they obtained carcasses with R conformation, and fatness degrees of 3+ and 3 for GP [38] and OC-fed animals [40,43], respectively. On the other hand, our carcasses were classified in category O+ and recorded a fatness level of 2, corresponding to carcasses with low-medium muscle development and a light fat coverage. In this sense, our values were lower than those obtained in previous studies employing GP and OC [38,40,43], though it could be attributed to different aspects, as the genotype, lower initial live weight, feeding management, among others [57]. Nevertheless, our results could be considered in line with the morphometric traits described in literature [19,44,45] for *Ovella Galega* breed. As previously mentioned, Galician lamb is in fact an autochthonous small size breed, not specialized for meat production, presenting light carcasses with an acceptable conformation and unelevated fatness levels [19,44,45]. Moreover, it is worth noting that the absence of significant differences in these subjective variables agrees with the aforementioned results about productive parameters and ultrasound subcutaneous fat thickness, where all treatments did not present statistically important variations in this investigation.

Thus, no significant (*p* > 0.05) changes were detected among groups for age, growth parameters, carcass weights, yields, carcass conformation, or fatness degree. This aspect may partially explain the absence of differences in carcass measurements (among treatments). In fact, no treatment-related differences (*p* > 0.05) were found for any of the evaluated parameters under the conditions of this study. Similarly, Araujo et al. [33] indicated that the inclusion of increasing levels of dehydrated BG, up to an 80% replacement, during 74 days did not influence carcass morphometric measures of Santa Ines lambs, except for croup circumference and thoracic width. In addition, other authors [36], using these same percentages of dried BG in Santa Ines lambs for 94 days, found similar carcass zoometry, apart from the internal carcass and leg lengths. Previous studies showed that also feeding lambs with diets containing up to 20% of GP did not alter carcass length [37,38] or leg length values [38]. In the same way, other researchers [58] found that supplementing with 280 g/d of OC during 98 days did not significantly change the carcass measures of Barberine lambs. However, these investigations [33,36,37,38,58] reported greater values than those found in the present work, which could be related to differences in initial live weight, breed, age, or feeding management, whereas our data resulted to be consistent with the range of values related to the internal carcass length (38.3–58.88 cm) and leg length (16.4–30.73 cm) found in the literature for *Ovella Galega* lambs and other light genotypes produced in Spain [19,49]. Otherwise, it is complicated to compare and discuss these outcomes, not only because of the limited number of studies on Galician lambs, but also because, in previous investigations, different breeds, ages, and feeding management were considered. Moreover, discrepancies may also appear due to the employ of distinct procedures for carcass morphometric evaluation [32,33,59,60].

The lack of statistical differences in the morphometric measures among dietary groups was also reflected in the carcass compactness indices, which did not show significant variations (*p* > 0.05) among animal lots, despite the incorporation of alternative ingredients in the diets. Other works found no effects on the CCI from the incorporation of dried BG [36], GP silage [38], or dried stoned OC [42] in ovine diets. In turn, Araujo et al. [33] observed that this variable diminished as the amount of employed BG increased, showing the lowest values starting from 60% feed replacement in Santa Ines lambs, whereas, considering the oil mill residue, it was seen that the supplementation with 280 g/d of crude OC improved the CCI in Barbarine lambs [58]. The results obtained in this study (0.14 ± 0.01 kg/cm) were lower than those observed in the above-mentioned investigations (0.19–0.30 kg/cm), using BG, GP, and OC as alternative dietary ingredients, although they were close to the range of values (0.12–0.14 kg/cm) seen by Fernández et al. [19] in Galician lambs.

Furthermore, considering the carcass fat thickness, GP10 recorded the greatest values (6.83 mm), followed by OC10 (6.28 mm), BG10 (5.70 mm), and CON (5.00 mm), although without significant differences (*p* > 0.05). This same trend (GP10 > OC10 > BG10 > CON) was observed also in the in vivo final SFT values, discussed above, also without significant variations. Overall, data showed that the dietary treatments did not significantly affect (*p* > 0.05) fat thickness, indicating that the inclusion of alternative ingredients did not produce substantial differences in the carcass lipid deposition under the conditions of this study. This finding is in agreement with previous studies where lambs were supplemented with BG [36], GP [38], and OC [42], and any significant changes were observed in this variable, whereas some authors [50] found increased values replacing (at 4, 8, 12, and 16% level) wheat straw with dried stoned OC. Examining the values obtained, our data were higher than those found in the above cited works [36,38,42,50], as well as in comparison with those reported in light lambs produced in Spain and sacrificed at a similar age [49]. Nonetheless, these investigations measured the subcutaneous fat thickness in different point of the *Longissimus thoracis and lumborum* muscle. As a consequence, our values are not comparable, since a different methodology was employed. Anyway, it is worth highlighting that both the in vivo measurements and the subjective and objective evaluation of the fat levels are in agreement, showing no statistically significant differences in the fatness degree values at slaughtering. Furthermore, in line with the morphometric results, no statistical evidence of treatment effects was found for body conformation and muscle development, a key aspect in production systems aimed to local markets and light lamb carcasses.

Finally, carcass pH values were also not influenced (*p* > 0.05) by the type of diet employed. The lack of a significant impact of the incorporation of BG, GP, and OC in lamb alimentation on this parameter is not uncommon. Indeed, in previous studies [34,37,42,53,54], it was observed that the use of these three by-products did not alter lamb carcass pH, even when incorporated at higher levels than those used in this study. These investigations reported values of 5.80 for lambs fed BG, 5.66–5.78 for fed GP, and 5.67–6.6 for the animals finished with OC-containing feed. These outcomes can be considered comparable to those obtained in the present study, ranging between 5.94 and 5.88. Furthermore, our data are within the generally acceptable pH range for ovine carcasses (5.50–5.98) [53,57,61], suggesting a normal post-mortem muscle acidification and absence of pre-slaughter stress [42,62].

### 3.2. Effect of the Finishing Diet on the Commercial Meat Cuts

Table 4 presents the data obtained by the dissection of the left half-carcass into commercial cuts of meat. As can be seen, these parameters were not significantly affected (*p* > 0.05) by the type of diet, showing comparable values among groups under the condition of the present study. This tendency was also detected in Polish Merino lambs finished with BG (35%) containing-feed, where carcass cuts reported similar percentages to those reported in the control group, except for the ribs, which recorded lower values in BG fed group [34]. Moreover, other investigators [63] observed that replacing the 11% of clove hay with dehydrated grape residue did not modify carcass cuts in goat kids. In addition, in agreement with our outcomes, other researchers [42] did not find significant variations in carcass meat portions from lambs supplemented with a 10% of dried stoned OC during 49 days, and the same tendency was observed with a higher level of residue (20%). The same trend was reported in a previous work [58], where Barbarine lambs supplemented with 280 g/animal/day of crude OC for 98 days. Besides, our findings are in line with the results previously discussed on productivity and carcass characteristics (as ADG, carcass weights, carcass yields, and morphometric attributes), where no significant dietary effects were detected.

As regards the percentages obtained, leg (25.62%) represented the most abundant commercial portion, followed by the ribs (20.40%) and shoulder (13.90%), while the neck (6.98%), loin (4.67%), breast (3.56%), and tenderloin (1.62%) occupied lower proportions. Although the leg is recognized to be the main cut in light lamb carcasses, the relative order of the subsequent portions (e.g., ribs and shoulder) could vary among studies [34,42,49,58], including those carried out on the same genotype [19,20,44]. These discrepancies could be related to different factors, such as slaughter age and feeding strategy, among others [57]. Nevertheless, they are especially and largely affected by the type of cutting practice employed [49,64]. These aspects, in fact, make it particularly complex to compare our data with those present in the literature and the discussion of singular cut proportions. Moreover, there is still a reduced number of investigations evaluating carcass cuts in Galician lambs [19,20,44] and in ovine supplemented with agri-food by-products, which limits direct comparisons among trials [34,42,58].

Despite these limitations, the distribution pattern of the main carcass cuts could have relevant commercial implications. In light lamb production systems, especially in traditional and local markets, carcass value is in fact mainly defined by the yield of the high-value cuts, such as the leg and shoulder. In the present work, these meat cuts occupied the largest proportions of the carcass and were not statistically (*p* > 0.05) influenced by the dietary treatment. Thus, the results obtained suggests that the use of BG, GP, and OC as partial concentrate replacers did not substantially change the commercial quality or market acceptability of the carcasses. This aspect is particularly important for local production systems as those involving *Ovella Galega* lambs, where animals are typically sold at low slaughter weights and are principally destined to regional consumption. In this context, the preservation of the expected carcass cuts yields is essential to maintain the product economic value.

Furthermore, the absence of statistically significant reduction in meat cut yields (especially in the high-priced cuts), along with the potential reduction of feed costs through the partial substitution of conventional concentrate ingredients with local agro-industrial by-products, may contribute to improving both the sustainability and profitability of small-scale production systems. In this sense, replacing 10% of concentrate with BG, GP, and OC in the finishing diets of *Ovella Galega* lambs could represent a viable approach that maintains carcass marketability while supporting resource valorization and circular economy practices.

## 4. Conclusions

Overall, the results of this study demonstrated that replacing 10% of the concentrate with brewers’ grain, grape pomace, or olive cake in the finishing diets of *Ovella Galega* lambs did not significantly compromise the growth parameters, carcass quality, or commercial cutting yields under the experimental conditions evaluated. In particular, the absence of statistically relevant changes in the percentages of the main commercial cuts, such as leg and shoulder, suggests that the use of these agri-food by-products could favor the maintenance of the carcass commercial value, relevant for the local light lamb markets.

Thus, the partial substitution of conventional concentrate with the evaluated agri-food residues agri-food residues could be a viable and sustainable feeding strategy for these autochthonous lambs. Moreover, this approach also represents an encouraging solution to diminish food waste, valorize local resources, and boost the circular economy in ovine production systems, in line with the current sustainability policies.

Furthermore, Galician lambs showed a good adaptation capacity to these alternative ingredients. In this sense, considering the limited information on the quality of this local breed as a meat producer, as well as the application of innovative dietary systems, this work could be considered a starting point to improve the handling of these endangered lambs. However, further investigations should be carried out to assess the effect of these local agro-industrial by-products on lamb meat quality and nutritional value, and to evaluate the economic implications, including effect size estimation and a detailed cost-benefit analysis.

## Figures and Tables

**Table 1:** Chemical composition of the dried brewers’ grain, grape pomace, and olive cake.

Parameters	BG	GP	OC
DM (%)	90.40	91.69	92.15
EE (% DM)	5.50	4.73	14.51
CP (% DM)	17.34	16.39	4.36
Ash (% DM)	4.07	9.74	4.46
CF (% DM)	12.49	24.28	34.37
ADF (% DM)	17.60	31.40	43.44
NDF (% DM)	35.30	34.81	55.00
Ca (mg/100 g)	118.38	252.36	125.24
P (mg/100 g)	286.56	280.3	159.08

BG: Brewers’ grain; GP: Grape pomace; OC: Olive cake; DM: Dry matter; EE: Ether extract; CP: Crude protein; CF: Crude fiber; ADF: Acid detergent fiber; NDF: Neutral detergent fiber.

**Table 2:** Ingredients (expressed as g/kg feed) and chemical composition of the finishing feeds allocated to the *Ovella Galega* lambs during the fattening period.

	Feeds			
	CON	BG10	GP10	OC10
**Ingredients (g/kg feed)**				
Barley	282	235	235	224
Soybean meal (44% CP)	211	199	202	232
Cracked corn	200	194	190	170
Wheat	120	90	90	100
Brewers’ grain	−	100	−	−
Grape pomace	−	−	100	−
Olive cake	−	−	−	100
Soybean hulls	70	70	70	70
Wheat bran	60	60	60	60
Calcium carbonate	17	17	17	17
Beet molasses	16	16	16	16
Soybean oil	15	10	11	2
Salt	6	6	6	6
Mineral–vitamin premix 0.3% ^1^	3	3	3	3
**Chemical composition**				
DM (%)	88.96	88.77	88.99	89.47
EE (% DM)	2.68	3.1	2.82	2.88
CP (% DM)	18.44	19.2	19.76	19.46
Ash (% DM)	5.23	5.35	5.25	5.7
CF (% DM)	5.39	6.35	6.96	6.97
ADF (% DM)	10.09	10.48	12.74	10.77
NDF (% DM)	37.07	31.5	27.29	29.08
Ca (mg/100 g)	216.84	247.32	283.01	298.06
P (mg/100 g)	329.54	341.5	383.39	287.03

CON: Control feed—conventional concentrate; BG10: Brewers’ grain feed—concentrate containing 10% of brewers’ grain; GP10: Grape pomace feed—concentrate containing 10% of grape pomace; OC10: Olive cake feed—concentrate containing 10% of olive cake. ^1^ Mineral–vitamin premix for lambs: 5000 IU/kg Vitamin A, 1400 IU/kg Vitamin D3, 8.00 mg/kg Vitamin E, 1.20 mg/kg I, 0.90 mg/kg Co, 64.00 mg/kg Mn, 75.08 mg/kg Zn, 0.20 mg/kg Se, 20.97 mg/kg Fe. DM: Dry matter; EE: Ether extract; CP: Crude protein; CF: Crude fiber; ADF: Acid detergent fiber; NDF: Neutral detergent fiber.

**Table 3:** Effect of the dietary treatment on the productive parameters, carcass measurements, and carcass pH from *Ovella Galega* lambs.

Parameters	Dietary Treatments				SEM	Sig.
	CON	BG10	GP10	OC10		
IBW (kg)	7.01	7.25	6.79	7.34	0.198	ns
BWS (kg)	12.70	12.47	12.96	13.15	0.182	ns
ADG (g/day)	87.29	91.89	101.98	87.95	3.551	ns
HCW (kg)	6.20	6.23	6.43	6.50	0.109	ns
CCW (kg)	5.95	5.90	6.15	6.20	0.098	ns
HCY (%)	48.91	49.82	49.55	49.34	0.394	ns
CCY (%)	46.93	47.25	47.47	47.07	0.351	ns
Conformation ^1^	5.25	5.50	6.13	5.75	0.139	ns
Fatness degree ^2^	5.13	4.63	4.63	4.75	0.125	ns
ECL (cm)	44.38	43.79	43.88	44.71	0.311	ns
ICL (cm)	42.89	43.14	42.25	43.79	0.397	ns
LL (cm)	29.69	30.25	30.10	30.69	0.245	ns
WS (cm)	9.07	9.71	9.94	9.89	0.169	ns
WB (cm)	11.19	10.94	11.09	11.48	0.194	ns
ACB (cm)	29.44	29.50	29.31	30.19	0.243	ns
PCB (cm)	30.06	29.56	30.00	30.06	0.205	ns
CT (cm)	49.69	48.81	49.75	50.25	0.304	ns
FT (mm)	5.00	5.70	6.83	6.28	0.312	ns
CCI (kg/cm)	0.14	0.14	0.15	0.14	0.002	ns
pH (24 h)	5.94	5.89	5.88	5.88	0.014	ns

SEM: Standard error of the mean; Sig: Significance; ns: Not significant; CON: Control feed group; BG10: Group supplemented with brewers’ grain feed; GP10: Group supplemented with grape pomace feed; OC10: Group supplemented with olive cake feed. IBW: initial live weight (at start of finishing diet); BWS = Live weight at slaughtering; ADG = Average daily gain; HCW = Hot carcass weight; CCW = Cold carcass weight; HCY: Hot carcass yield; CCY: Cold carcass yield; ECL: External carcass length; ICL: Internal carcass length; LL: Leg length; WS: Width of shoulders; WB: Width of buttocks; ACB: Anterior circumference of buttocks; PCB: Posterior circumference of buttocks; CT: Circumference of thorax; FT: Fat thickness; CCI: Carcass compactness index. ^1^ SEUROP conformation subclasses, in ascending order (P− to S+), were numerically coded from 1 to 18, respectively; ^2^ Fatness subclasses, in ascending order (1− to 5+), were numerically coded from 1 to 15, respectively.

**Table 4:** Effect of the dietary treatment on the main commercial meat cuts (expressed as left half-carcass %) from *Ovella Galega* lambs.

Meat Cuts (Half Carcass %)	Dietary Treatments				SEM	Sig.
	CON	BG10	GP10	OC10		
Leg	25.42	26.62	25.03	25.40	0.319	ns
Ribs	20.36	20.85	20.26	20.13	0.333	ns
Loin	4.67	4.49	5.07	4.45	0.101	ns
Breast	4.05	3.72	3.60	2.87	0.164	ns
Tenderloin	1.53	1.58	1.65	1.71	0.052	ns
Shoulder	14.13	14.20	13.56	13.73	0.164	ns
Neck (total)	6.68	6.74	7.34	7.15	0.165	ns

SEM: Standard error of the mean; Sig: Significance; ns: Not significant; CON: Control feed group; BG10: Group supplemented with brewers’ grain feed; GP10: Group supplemented with grape pomace feed; OC10: Group supplemented with olive cake feed.

## Data Availability

The raw data supporting the conclusions of this article will be made available by the authors upon request.

## References

[1] Billi M., Marchant G., Bustamante G. (2022). Sustainability as a Meta-Narrative: The Semantics of Global Governance? A Systems-Theoretical and Concept-Historical Analysis. Econ. Política.

[2] UN United Nations Transforming Our World: The 2030 Agenda for Sustainable Development. https://www.un.org/en/development/desa/population/migration/generalassembly/docs/globalcompact/A_RES_70_1_E.pdf.

[3] Belardi I., De Francesco G., Alfeo V., Bravi E., Sileoni V., Marconi O., Marrocchi A. (2025). Advances in the Valorization of Brewing By-Products. Food Chem..

[4] UN (2024). United Nations Department for Economic and Social Affairs. World Population Prospects 2024: Summary of Results.

[5] Čolović D., Rakita S., Banjac V., Đuragić O., Čabarkapa I. (2019). Plant Food By-Products as Feed: Characteristics, Possibilities, Environmental Benefits, and Negative Sides. Food Rev. Int..

[6] UN (2024). United Nations Environment Programme. Food Waste Index Report 2024.

[7] Jalal H., Giammarco M., Lanzoni L., Akram M.Z., Mammi L.M.E., Vignola G., Chincarini M., Formigoni A., Fusaro I. (2023). Potential of Fruits and Vegetable By-Products as an Alternative Feed Source for Sustainable Ruminant Nutrition and Production: A Review. Agriculture.

[8] Correddu F., Lunesu M.F., Buffa G., Atzori A.S., Nudda A., Battacone G., Pulina G. (2020). Can Agro-Industrial by-Products Rich in Polyphenols Be Advantageously Used in the Feeding and Nutrition of Dairy Small Ruminants?. Animals.

[9] FAO. Food and Agriculture Organization of the United Nations FAOSTAT 2026. https://www.fao.org/faostat/es/#data/QCL.

[10] MAPA (2024). Ministerio de Agricultura Pesca y Alimentación. Anuario de Estadística 2024.

[11] Chowdhary P., Gupta A., Gnansounou E., Pandey A., Chaturvedi P. (2021). Current Trends and Possibilities for Exploitation of Grape Pomace as a Potential Source for Value Addition. Environ. Pollut..

[12] Obeidat B.S., Kridli R.T. (2021). Olive Cake in Livestock Nutrition. Jordan J. Agric. Sci..

[13] Belardi I., Marrocchi A., Alfeo V., Sileoni V., De Francesco G., Paolantoni M., Marconi O. (2023). Sequential Extraction and Attenuated Total Reflection–Fourier Transform Infrared Spectroscopy Monitoring in the Biorefining of Brewer’s Spent Grain. Molecules.

[14] Almanza-Oliveros A., Bautista-Hernández I., Castro-López C., Aguilar-Zárate P., Meza-Carranco Z., Rojas R., Michel M.R., Martínez-Ávila G.C. (2024). Grape Pomace—Advances in Its Bioactivity, Health Benefits, and Food Applications. Foods.

[15] Yang K., Qing Y., Yu Q., Tang X., Chen G., Fang R., Liu H. (2021). By-Product Feeds: Current Understanding and Future Perspectives. Agriculture.

[16] MAPA Ministerio de Agricultura Pesca y Alimentación Datos Censales de La Raza *Ovella Galega*. https://servicio.mapa.gob.es/arca2/censos/consulta/4139.

[17] Adán S., García J., Domínguez B., Justo J.R., Lama J., Fernández M., Rivero C.J., Rois D. (2007). Estudio Del Crecimiento de Los Corderos de La Raza *Ovella Galega*. Arch. Zootec..

[18] Adán S., Béjar D. (2011). Avance de Resultados de Parámetros de Crecimiento y Canal de Corderos de *Ovella Galega* Sacrificados a 4 Meses. Actas Iberoam. Conserv. Anim..

[19] Fernández M., Adán S., Domínguez B., Rivero C.J., Justo J.R., Arias A., García Fontán M.C., Lorenzo J.M., Lama J.J., López C. (2011). Parámetros de Crecimiento y de La Canal de Corde Ros de La Raza *Ovella Galega* Sacrificados a 45 Días. Arch. Zootec..

[20] Luaces M.L., Calvo C., Fernández A., Viana J.L., Fernández B., Sánchez L. (2007). Estudio de Las Piezas Comerciales y Su Desarrollo En Canales de Corderos de La Raza Ovina Gallega. Arch. Zootec..

[21] Cittadini A., Bermúdez R., Domínguez R., Pateiro M., Lorenzo J.M. (2025). Orujo de oliva, bagazo de cerveza y de uva: Tres valiosos subproductos agroalimentarios como fuentes de alimentación alternativa en el ganado ovino. Eurocarne Rev. Int. Sect. Cárnico.

[22] AOAC (2007). Official Methods of Analysis.

[23] Van Soest P.J., Robertson J.B., Lewis B.A. (1991). Methods for Dietary Fiber, Neutral Detergent Fiber, and Nonstarch Polysaccharides in Relation to Animal Nutrition. J. Dairy Sci..

[24] Santos A., Giráldez F.J., Mateo J., Frutos J., Andrés S. (2018). Programming Merino Lambs by Early Feed Restriction Reduces Growth Rates and Increases Fat Accretion during the Fattening Period with No Effect on Meat Quality Traits. Meat Sci..

[25] Esquivelzeta C., Casellas J., Fina M., Campo M.d.M., Piedrafita J. (2017). Carcass Traits and Meat Fatty Acid Composition in Mediterranean Light Lambs. Can. J. Anim. Sci..

[26] Ripoll G., Joy M., Alvarez-Rodriguez J., Sanz A., Teixeira A. (2009). Estimation of Light Lamb Carcass Composition by in Vivo Real-Time Ultrasonography at Four Anatomical Locations. J. Anim. Sci..

[27] Teixeira A., Matos S., Rodrigues S., Delfa R., Cadavez V. (2006). In Vivo Estimation of Lamb Carcass Composition by Real-Time Ultrasonography. Meat Sci..

[28] Afonso J., Guedes C.M., Teixeira A., Santos V., Azevedo J.M.T., Silva S.R. (2019). Using Real-Time Ultrasound for in Vivo Assessment of Carcass and Internal Adipose Depots of Dairy Sheep. J. Agric. Sci..

[29] European Commission (2009). Council Regulations (EC) No 1099/2009 of 24 September 2009 on the Protection of Animals at the Time of Killing. Off. J. Eur. Union..

[30] Gurgel A.L.C., Difante G., Neto J.V.E., de Araújo C.G., Costa M.G., Ítavo L.C., de Araujo I.M., Costa C.M., Santana J.C., Ítavo C.C. (2021). Prediction of Carcass Traits of Santa Inês Lambs Finished in Tropical Pastures through Biometric Measurements. Animals.

[31] European Commission (2008). Reglamento (CE) no 1249/2008 de la Comisión, de 10 de Diciembre de 2008, Por El Que Se Establecen Disposiciones de Aplicación Relativas a los Modelos Comunitarios de Clasificación de Las Canales de Vacuno, Porcino y Ovino y a La Comunicación de Sus Precios.

[32] Teixeira A., Cadavez V., Delfa R., Bueno M.S. (2004). Carcass Conformation and Joints Composition of Churra Galega Bragançana and Crossbred Lambs by Suffolk and Merino Precoce Sire Breeds. Spanish J. Agric. Res..

[33] de Araújo M.S., Pimentel P.G., Batista A.S.M., Moreira G.R., Pinto A.P. (2021). Biometric and Carcass Measurements in Santa Ines Lambs Fed Dehydrated Brewery Residue. Rev. Cienc. Agron..

[34] Radzik-Rant A., Rant W., Niznikowski R., Swiatek M., Szymanska Ż., Slezak M., Orlowski E., Bednarczyk M., Morales-Vilavicencio A. (2019). The Effect of the Brewing and Milling By-Products Containing in the Lambs Diet on Body Weight Growth, Slaughter Value and Meat Quality. Ann. Warsaw Univ. Life Sci. SGGW Anim. Sci..

[35] Castillo D., Villar L., Cancino K., Caballero V., Odeón M., Ferrari J., Villagra S. (2021). Podemos Engordar Corderos con Bagazo de Cerveza?: Un Subproducto Con Alto Contenido Proteico y Disponible A La Vuelta de la Esquina.

[36] Cavilhão C., Costa P.B., Vilela C.G., Karvatte Junior N., Hermes P.R., Taffarel L.E. (2013). Avaliação in Vivo e Características Da Carcaça de Cordeiros Santa Inês Alimentados Com Resíduo de Cervejaria. Sci. Agrar. Parana..

[37] Antunović Z., Šalavardić Ž.K., Steiner Z., Đidara M., Drenjančević M., Ronta M., Pavić V., Barron L.J., Novoselec J. (2023). Meat Quality, Metabolic Profile and Antioxidant Status of Lambs Fed on Seedless Grape Pomace (*Vitis vinifera* L.). Ann. Anim. Sci..

[38] Massaro F.L., Bumbieris V.H., Pereira E.S., Zanin E., Horst E.H., Calixto O.P., Peixoto E.L., Galbeiro S., Mizubuti I.Y. (2022). Grape Pomace Silage on Growth Performance, Carcass, and Meat Quality Attributes of Lambs. Sci. Agric..

[39] Yao Y., Wang H., Lu Z., Nian F., Zheng C., Li F., Tang D. (2023). Improving Shelf Life and Content of Unsaturated Fatty Acids in Meat of Lambs Fed a Diet Supplemented with Grape Dregs. Foods.

[40] Benbati M., Elfazazi K., Chafki L., Azzouzi H., Haddioui A., El Hansali M., Keli A. (2022). Effects of Olive Cake on Fattening Performances, Carcass Characteristics and Meat Quality of Lambs. Afr. Mediterr. Agric. J..

[41] Mioč B., Pavić V., Vnučec I., Prpić Z., Kostelić A., Sušić V. (2007). Effect of Olive Cake on Daily Gain, Carcass Characteristics and Chemical Composition of Lamb Meat. Czech J. Anim. Sci..

[42] Sucu E., Akbay K.C., Şengül Ö., Yavuz M.T., Ibrahim A.K. (2018). Effects of Stoned Olive Pomace on Carcass Characteristics and Meat Quality of Lambs. Turk. J. Vet. Anim. Sci..

[43] Benbati M., Belafqih B., El Otmani S., Mounsif M., Keli A. (2014). Effect of the Level of Incorporation of Olive Cake in the Diet on Lamb Fattening Performance and Carcass Characteristics. Forage Resources and Ecosystem Services Provided by Mountain and Mediterranean Grasslands and Rangelands.

[44] Sanchez L., Fernandez B., Lopez M., Sanchez B. (2000). Caracterización Racial y Orientaciones Productivas de La Raza Ovina Gallega. Arch. Zootec..

[45] MAPA Ministerio de Agricultura Pesca y Alimentación Raza *Ovella Galega*. https://www.mapa.gob.es/es/ganaderia/temas/zootecnia/razas-ganaderas/razas/catalogo-razas/ovino/gallega/default.aspx.

[46] Blanco C., Giráldez F.J., Prieto N., Benavides J., Wattegedera S., Morán L., Andrés S., Bodas R. (2015). Total Mixed Ration Pellets for Light Fattening Lambs: Effects on Animal Health. Animal.

[47] Cheng X., Du X., Liang Y., Degen A.A., Wu X., Ji K., Gao Q., Xin G., Cong H., Yang G. (2023). Effect of Grape Pomace Supplement on Growth Performance, Gastrointestinal Microbiota, and Methane Production in Tan Lambs. Front. Microbiol..

[48] Bahrami Y., Foroozandeh A.D., Zamani F., Modarresi M., Eghbal-Saeid S., Chekani-Azar S. (2010). Effect of Diet with Varying Levels of Dried Grape Pomace Digestibility and Growth Performance Lambs. J. Anim. Plant Sci..

[49] Miguélez E., Zumalacárregui J.R., Osorio M.A., Mateo J. (2007). Características de La Canal de Cordeo Lechal de Diversas Razas Producidas En España (Revisión Bibliográfica). ITEA.

[50] Salih S., Mahmood A. (2023). Effect of Olive Pomace as Supplement on Growth, Carcass and Meat Characteristics of Karadi Lambs. Indian J. Anim. Sci..

[51] Ripoll G., Joy M., Sanz A. (2010). Estimation of Carcass Composition by Ultrasound Measurements in 4 Anatomical Locations of 3 Commercial Categories of Lamb. J. Anim. Sci..

[52] Zhao J.X., Li Q., Zhang R.X., Liu W.Z., Ren Y.S., Zhang C.X., Zhang J.X. (2018). Effect of Dietary Grape Pomace on Growth Performance, Meat Quality and Antioxidant Activity in Ram Lambs. Anim. Feed Sci. Technol..

[53] Chikwanha O.C., Muchenje V., Nolte J.E., Dugan M.E.R., Mapiye C. (2019). Grape Pomace (*Vitis Vinifera* L. Cv. Pinotage) Supplementation in Lamb Diets: Effects on Growth Performance, Carcass and Meat Quality. Meat Sci..

[54] Ozdogan M., Ustundag A.O., Yarali E. (2017). Effect of Mixed Feeds Containing Different Levels of Olive Cake on Fattening Performance, Carcass, Meat Quality and Fatty Acids of Lambs. Trop. Anim. Health Prod..

[55] Wang W., Zhang X., Wei H., Wang S., Ye Y., He L., Zhang K., Lu Y., Zhang Z., Huang Y. (2024). Effects of Feeding Systems on the Growth Performance, Carcass Characteristics, and Meat Quality in Sheep: A Meta-Analysis. Animals.

[56] Calderón-Cortés J.F., González-Vizcarra V.M., Pétriz-Celaya Y., Pujol L.C., Barreras A., Plascencia A. (2018). Energy Value of Unfermented Dried Grape Pomace as Substitute of Alfalfa Hay in Diets for Growing Lambs. Austral J. Vet. Sci..

[57] Lorenzo J.M., Munekata P.E.S., Barba F.J., Toldrá F. (2019). More than Beef, Pork and Chicken—The Production, Processing, and Quality Traits of Other Sources of Meat for Human Diet.

[58] Hamdi H., Majdoub-Mathlouthi L., Picard B., Listrat A., Durand D., Znaïdi I.A., Kraiem K. (2016). Carcass Traits, Contractile Muscle Properties and Meat Quality of Grazing and Feedlot Barbarine Lamb Receiving or Not Olive Cake. Small Rumin. Res..

[59] Majdoub-Mathlouthi L., Saïd B., Say A., Kraiem K. (2013). Effect of Concentrate Level and Slaughter Body Weight on Growth Performances, Carcass Traits and Meat Quality of Barbarine Lambs Fed Oat Hay Based Diet. Meat Sci..

[60] Osório J.C.S., Osório M.T.M. (2005). Sheep Meat Production. Vivo and Carcass Evaluation Techniques.

[61] Jiang H., Wang Z., Ma Y., Qu Y., Lu X., Guo H., Luo H. (2014). Effect of Dietary Lycopene Supplementation on Growth Performance, Meat Quality, Fatty Acid Profile and Meat Lipid Oxidation in Lambs in Summer Conditions. Small Rumin. Res..

[62] Duarte M.S., Paulino P.V.R., Fonseca M.A., Diniz L.L., Cavali J., Serão N.V.L., Gomide L.A.M., Reis S.F., Cox R.B. (2011). Influence of Dental Carcass Maturity on Carcass Traits and Meat Quality of Nellore Bulls. Meat Sci..

[63] Awadalla I., Mohamed M.I., Soliman A.A.M., Shoukry M.M. (2003). Utilization of Grape Marc in Formulating Rations for Growing Goat Kids. J. Anim. Poult. Prod..

[64] Colomer Rocher F., Morand-Fehr P., Kirton A.H., Delfa Belenguer R., Sierra Alfranca I. (1988). Metodos Normalizados Para El Estudio de Los Caracteres Cuantitativos y Cualitativos de Las Canales Caprinas y Ovinas.

